# Prognostic analysis of stage IIIC1p cervical cancer patients

**DOI:** 10.3389/fonc.2024.1362281

**Published:** 2024-04-25

**Authors:** Ting Gao, Zixuan Yang, Liqun Wei, Xiaobi Tang, Shanshan Ma, Li Jiang, Yong Zhang, Fang Wu

**Affiliations:** Department of Radiation Oncology, The First Affiliated Hospital of Guangxi Medical University, Nanning, China

**Keywords:** cervical cancer, stage IIIC1p, overall survival, adjuvant chemotherapy, lymph node metastasis

## Abstract

**Background:**

Stage IIIC1p cervical cancer is characterized by marked heterogeneity and considerable variability in the postoperative prognosis. This study aimed to identify the clinical and pathological characteristics affecting the survival of patients diagnosed with stage IIIC1p cervical cancer.

**Methods:**

We retrospectively analyzed patients diagnosed with stage IIIC1p cervical cancer who underwent radical hysterectomy and lymph node dissection between March 2012 and March 2022. Overall survival (OS) was estimated using Kaplan-Meier survival curves. Univariate and multivariate Cox proportional hazards models were used to evaluate prognostic factors for OS and forest plots were used to visualize these findings. Nomogram charts were created to forecast survival rates at 3 and 5 years, and the accuracy of predictions was evaluated using Harrell’s concordance index (C-index) and calibration curves.

**Results:**

The study cohort comprised 186 women diagnosed with stage IIIC1p cervical cancer. The median follow-up duration was 51.1 months (range, 30-91 months), and the estimated 5-year OS rate was 71.5%. Multivariate analysis revealed that concurrent chemoradiotherapy plus adjuvant chemotherapy (CCRT + AC), monocyte-lymphocyte ratio (MLR), ratio of lymph node metastasis (LNM), and squamous cell carcinoma antigen (SCCA) levels independently predicted OS.

**Conclusions:**

Significant prognostic disparities exist among patients diagnosed with stage IIIC1p cervical cancer. MLR, ratio of LNM, and SCCA were associated with poor OS. In contrast, the CCRT + AC treatment regimen appeared to confer a survival advantage.

## 1 Introduction

Cervical cancer is one of the most prevalent malignancies of the female reproductive system. According to 2020 worldwide cancer data, approximately 604,000 new cases and 342,000 fatalities occur annually, positioning it as the primary cause of non-breast gynecological malignancies (1). The occurrence and fatality rates of cervical cancer in China account for 18.3% and 17.6%, respectively, of the worldwide total. Despite a decreasing trend in incidence due to widespread early screening, incidence rates among younger patients are still on the rise (1–3).

Lymph node metastasis (LNM) significantly affects the cervical cancer prognosis. Radical hysterectomy and lymph node dissection are crucial for patients during the early stages. However, based on existing randomized controlled trials, we found insufficient evidence to suggest that hysterectomy improves the survival of women with locally advanced cervical cancer treated with either radiotherapy or chemoradiotherapy (4). The chances of pelvic LNM in cervical cancer stages IB2, IIA, and IIB were 11%, 13%, and 16%, respectively (5–7). After the 2018 International Federation of Gynecology and Obstetrics (FIGO) update, patients who were pathologically confirmed to have pelvic LNM were specifically labeled as stage IIIC1p. According to the US SEER Program, patients with stage IIIC1 disease have a higher disease-specific survival rate than patients with stage IIIA-IIIB disease. Additionally, the survival rate within the stage IIIC1 group decreased as the T stage increased (5-year overall survival [OS] rates were 74.8% for T1, 58.7% for T2, and 39.3% for T3) (8). This suggests that the outlook for individuals with cervical cancer and pelvic LNM varies owing to factors beyond the status of the lymph nodes. Hence, a more in-depth examination of the clinical and pathological characteristics of individuals diagnosed with stage IIIC1p cervical cancer may improve prognostic evaluation and personalized supplementary therapy.

Several studies have suggested that parameters such as the number of LNM, ratio of LNM (proportion of positive to total lymph nodes removed), and number of LNM sites are correlated with OS (9–11). Furthermore, the prognosis of patients with IIIC1p was found to be associated with pathological type (12, 13), pT2b (12), and neutrophil-to-lymphocyte ratio (NLR) > 3.8 (9). Nevertheless, elucidating which factors are more effective in assessing the outlook of individuals with IIIC1p cervical cancer remains unresolved. Therefore, the objective of this study was to identify the risk factors impacting prognosis, providing a basis for accurate prognostic assessment, and tailored therapeutic strategies to improve the survival and quality of life of patients with stage IIIC1p cervical cancer.

## 2 Materials and methods

### 2.1 Patients

A retrospective analysis was conducted on 186 patients with cervical cancer who had undergone radical hysterectomy and lymph node dissection at the First Affiliated Hospital of Guangxi Medical University, spanning the period from March 2012 to March 2022. Following the 2018 FIGO criteria, all patients with positive pelvic lymph nodes were re-staged to cervical cancer stage IIIC1p and underwent postoperative concurrent chemoradiotherapy (CCRT). The following criteria were used for exclusion (1): chemotherapy before surgery or radiotherapy (2), LNM in the para-aortic region (3), concurrent presence of other tumors (4), distant metastasis, and (5) no follow-up data. In this study, we collected data from patients with stage IIIC1p cervical cancer regarding LNM (including common iliac lymph node, number of LNM sites, the number and ratio of LNM), para-aortic lymph nodes (PALN) resection, vaginal brachytherapy utilization, type of radicality, postoperative high-risk factors (surgical margins and parametrial infiltration), intermediate-risk factors (tumor size, lymph-vascular space invasion [LVSI], depth of stromal invasion [DSI]), preoperative tumor markers (squamous cell carcinoma antigen [SCCA] and carcinoembryonic antigen [CEA]), adjuvant treatment regimens (CCRT and CCRT+ adjuvant chemotherapy [AC]), monocyte-to-lymphocyte ratio (MLR), and chemotherapy protocol. According to the literature, we classified LVSI positivity into focal and diffuse. Focal was defined as a single focus of LVSI recognized around a tumor, and diffuse was defined as diffuse LVSI (more than 1) recognized around the tumor (14). The study was approved by the institutional ethics review committee and was conducted in compliance with the principles of the Declaration of Helsinki (approval number: 2023-E724-01). Follow-up was concluded on October 10, 2023, via telephone interviews or outpatient medical records.

### 2.2 Treatment

All patients underwent radical hysterectomy and systematic bilateral pelvic lymphadenectomy. If common iliac lymph nodes were identified as being positive by the intraoperative frozen section or the PALN were identified as suspicious by visualization and palpation, para-aortic lymphadenectomy was also performed during radical surgeries. After surgery, external-beam radiation therapy to the pelvis was conducted on all patients. Extended field radiation with the upper margin up to T12-L1 was performed in common iliac lymph node-positive patients. The dose to the whole pelvis was 45-50.4 Gy at 25-28 fractions delivered five times per week. Patients with positive vaginal margins also received intracavitary brachytherapy with a dose of 30 Gy delivered in five fractions twice per week. The concurrent chemotherapeutic drugs included cisplatin, 5-fluorouracil, and paclitaxel. AC drugs included cisplatin, docetaxel, and paclitaxel.

### 2.3 Statistical analysis and study endpoints

The data were analyzed using SPSS 26.0, and R version 4.3.1. Quantitative variables were summarized using medians (P25, P75) or means ± standard deviations, and intergroup comparisons were made using the independent sample *t*-test or Mann-Whitney U test. The chi-square test was used to compare categorical variables, which were expressed as counts (%). To determine the prognostic factors for OS, we utilized the R programming language and conducted Cox regression analysis with the assistance of the ‘survival’ and ‘survminer’ packages. The results were presented as forest plots. Nomograms were constructed, and their predictive accuracies were assessed using Harrell’s concordance index (C-index) and 1,000 bootstrap resampling for the calibration curves.

The primary outcome measure was OS, which was defined as the period from the surgical procedure until either death or the most recent follow-up available.

## 3 Results

### 3.1 Patient clinicopathological characteristics

Our study included 186 patients diagnosed with stage IIIC1p cervical cancer. **Table 1** shows the clinicopathological characteristics of these patients. The mean patient age was 47 years. The vast majority of patients (87.60%) underwent a type C radical hysterectomy. The most prevalent histologic type was squamous cell carcinoma, accounting for 81.70% of cases. Non-squamous cell carcinomas included adenocarcinomas, adenosquamous carcinomas, and neuroendocrine carcinomas. Most cases showed tumor sizes ≤ 4 cm (80.10%), diffuse positive LVSI (52.10%), DSI >1/2 (81.20%), negative resection margins (94.60%), negative parametrial invasion (97.80%), negative common iliac lymph node (96.80%), 1-2 LNM (72.60%), LNM in one site (64.00%), no PALN resection (73.10%), and no vaginal brachytherapy utilization (68.80%).

**Table 1:** Patients’ characteristics.

Characteristics(n=186)	value
**Age**(years)	47.11 ± 9.15
Radicality of surgery	
Type A	16(8.60%)
Type B	7(3.80%)
Type C	163(87.60%)
Pathological type	
squamous cell carcinoma	152(81.70%)
non-squamous cell carcinoma	34(18.30%)
Tumor size	
≤4 cm	149(80.10%)
>4 cm	37(19.90%)
LVSI	
Diffuse	97(52.10%)
Focal	15(8.10%)
Negative	74(39.80%)
DSI	
≤1/2	35(18.80%)
>1/2	151(81.20%)
Surgical margins	
Positive	10(5.40%)
Negative	176(94.60%)
PI	
Positive	4(2.20%)
Negative	182(97.80%)
Common iliac lymph node	
Positive	6(3.20%)
Negative	180(96.80%)
Number of LNM	
**1-2**	135(72.60%)
**>2**	51(27.40%)
Number of LNM sites	
1	119(64.00%)
2	66(35.50%)
3	1(0.50%)
PALN resection	
Yes	50(26.90%)
No	136(73.10%)
Vaginal brachytherapy utilization	
Yes	58(31.20%)
No	128(68.80%)
Adjuvant therapy	
CCRT	117(62.90%)
CCRT+AC	69(37.10%)
CC protocol	
cisplatin	162(87.10%)
TP	14(7.50%)
FP	10(5.40%)
AC protocol	
TP	44(63.80%)
DP	25(36.20%)
**Ratio of LNM**	12.50%(7.14%-20.35%)
**SCCA** (ng/ml)	2.05(0.80-5.30)
**CEA** (ng/ml)	1.99(1.29-3.68)
**MLR**	0.26(0.20-0.32)

LVSI, lymph-vascular space invasion; DSI, depth of stromal invasion; PI, Parametrial infiltration; LNM, Lymph node metastasis; PALN, para-aortic lymph nodes; SCCA, squamous cell carcinoma antigen; CEA, carcinoembryonic antigen; MLR, monocyte-to-lymphocyte ratio; CCRT, concurrent chemoradiotherapy; CCRT+AC, concurrent chemoradiotherapy + adjuvant chemotherapy; CC, concurrent chemoradiotherapy; TP, cisplatin plus paclitaxel; FP, cisplatin plus 5-fluorouracil; DP, cisplatin plus docetaxel.

For non-normally distributed data, the median values for the number of LNM, ratio of LNM, SCCA, CEA, and MLR were 1, 12.50%, 2.05 ng/mL, 1.99 ng/mL, and 0.26, respectively. All patients received CCRT postoperatively, and 37.10% (n=69) received AC. In the concurrent chemotherapy regimens, weekly cisplatin, TP (cisplatin plus paclitaxel), and FP (cisplatin plus 5-fluorouracil) accounted for 87.1%, 7.5%, and 5.4% of treatments, respectively. For the AC regimen, TP and DP (cisplatin plus docetaxel) accounted for 63.8% and 36.2% of the treatments, respectively. No statistically significant differences in the patient characteristics between the CCRT and CCRT+AC treatment groups were observed (**Table 2**).

**Table 2:** Patient characteristics in different adjuvant therapy.

	CCRT	CCRT+AC	P value
**Age**	47.53 ± 8.93	46.41 ± 9.54	0.46
**Radicality of Surgery**			0.28
Type A	13(81.20%)	3(18.80%)	
Type B	4(57.10%)	3(42.90%)	
Type C	100(61.30%)	63(38.70%)	
**Pathological type**			0.81
squamous cell carcinoma	95(62.50%)	57(37.50%)	
non-squamous cell carcinoma	22(21.40%)	12(12.60%)	
**Tumor size**			0.78
≤4 cm	93(62.40%)	56(37.60%)	
>4 cm	24(64.90%)	13(35.10%)	
**LVSI**			0.70
Diffuse	61(62.90%)	36(37.15%)	
Focal	8(53.30%)	7(46.70%)	
Negative	48(64.90%)	26(35.10%)	
**DSI**			0.25
≤1/2	25(71.40%)	10(28.60%)	
>1/2	92(60.90%)	59(39.10%)	
**Surgical margins**			0.85
Positive	6(62.90%)	4(40.00%)	
Negative	111(63.10%)	65(36.90%)	
**PI**			0.59
Positive	2(50.00%)	2(50.00%)	
Negative	115(63.20%)	67(36.80%)	
**Common iliac lymph node**			0.85
Positive	4(66.70%)	2(33.30%)	
Negative	113(62.80%)	67(37.20%)	
**Number of LNM**			0.32
**1-2**	82(60.70%)	53(50.10%)	
**>2**	35(68.60%)	16(31.40%)	
**Number of LNM sites**			0.74
1	75(63.00%)	44(37.00%)	
2	41(62.10%)	25(37.90%)	
3	1(100.00%)	0(0.00%)	
**PALN resection**			0.24
Yes	28(56.00%)	22(44.00%)	
No	89(65.40%)	47(34.60%)	
**Vaginal brachytherapy utilization**			0.25
Yes	33(56.90%)	25(43.10%)	
No	84(65.60%)	44(34.40%)	
**Ratio of LNM**	12.50%(7.42%-20.71%)	12.50%(6.90%-21.11%)	0.78
**SCCA(ng/ml)**	1.70(0.75-5.15)	2.20(1.00-8.45)	0.19
**CEA(ng/ml)**	1.89(1.23-3.16)	2.22(1.30-3.79)	0.57
**MLR**	0.26(0.20-0.34)	0.24(0.19-0.32)	0.43

LVSI, lymph-vascular space invasion; DSI, depth of stromal invasion; PI, Parametrial infiltration; LNM, Lymph node metastasis; PALN, para-aortic lymph nodes; SCCA, squamous cell carcinoma antigen; CEA, carcinoembryonic antigen; MLR, monocyte-to-lymphocyte ratio.

### 3.2 Univariate and multivariate analyses for OS

**Figure 1:**
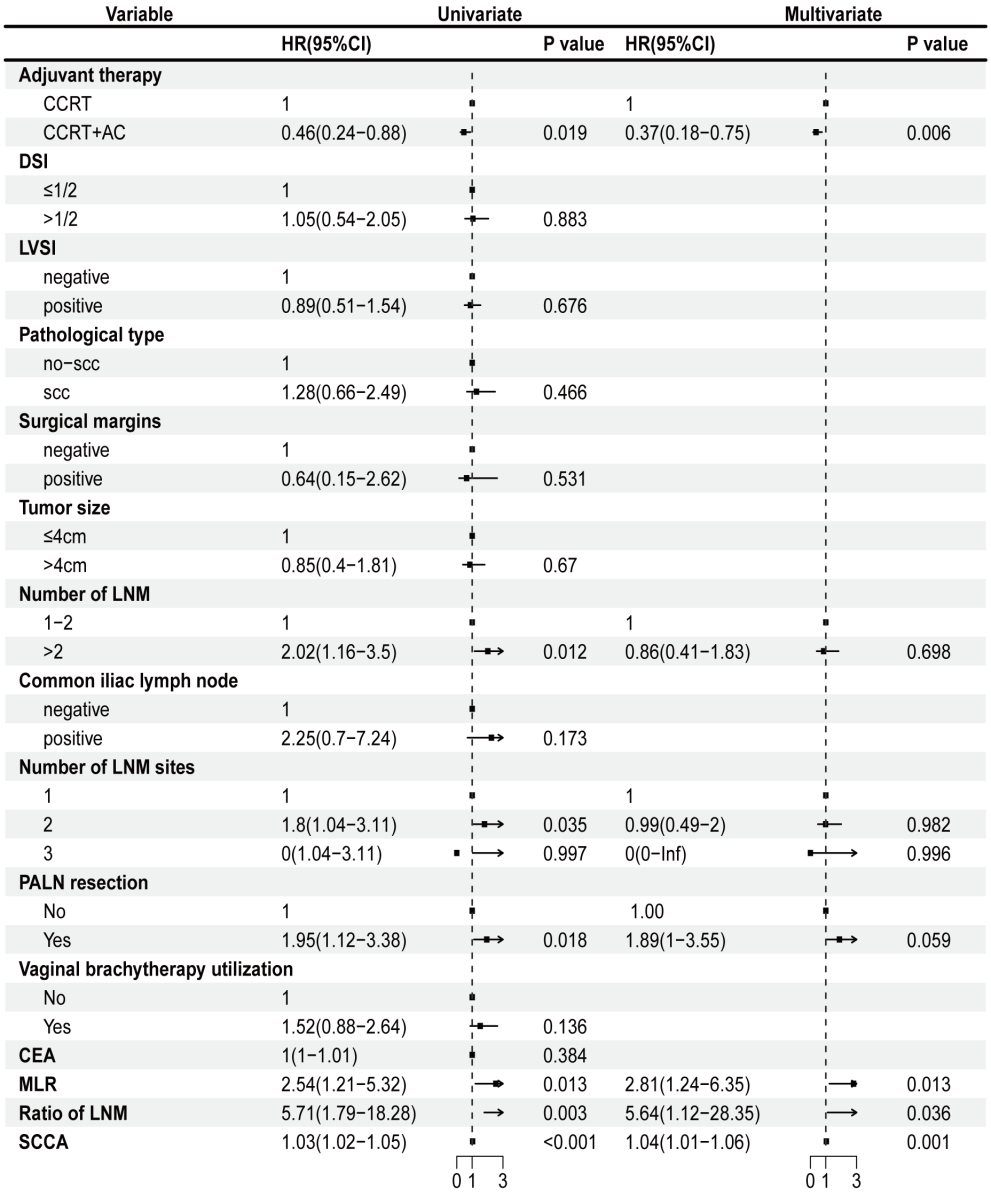
Univariate Cox regression analysis and multivariate Cox regression regarding OS. CCRT, concurrent chemoradiotherapy; CCRT+AC, concurrent chemoradiotherapy + adjuvant chemotherapy; DSI, depth of stromal invasion; LVSI, lymph-vascular space invasion; scc, squamous cell carcinoma; CEA, carcinoembryonic antigen; MLR, monocyte-to-lymphocyte ratio; LNM, Lymph node metastasis; PALN, para-aortic lymph nodes; SCCA, squamous cell carcinoma antigen; OS, overall survival.

**Figure 2:**
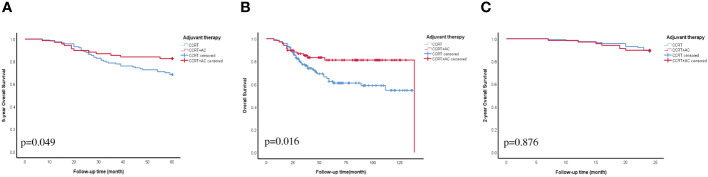
OS curve for adjuvant therapy. **(A)** 5-year OS. **(B)** OS. **(C)** 2-year OS. Abbreviations: CCRT, concurrent chemoradiotherapy; CCRT+AC, concurrent chemoradiotherapy + adjuvant chemotherapy; OS, overall survival.

We conducted univariate Cox regression analysis of the factors that could affect the outlook of individuals diagnosed with stage IIIC1p cervical cancer. These findings indicated that the combination of CCRT + AC, number of LNM >2, LNM in two site, PALN were resected, MLR, ratio of LNM, and SCCA were strongly linked to OS in individuals diagnosed with stage IIIC1p cervical cancer (p<0.05) (**Figure 1**). Multivariate Cox regression analysis further delineated CCRT + AC (hazard ratio [HR] =0.37, 95% confidence interval [CI] 0.18-0.75, p=0.006), MLR (HR=2.81, 95% CI 1.24-6.35, p=0.013), ratio of LNM (HR=5.64, 95% CI 1.12-28.35, p=0.036), and SCCA (HR=1.04, 95% CI 1.01-1.06, p=0.001) as independent prognostic factors for OS (**Figure 1**). Based on multivariate analysis, the Kaplan-Meier survival curves indicated that the CCRT + AC treatment regimen resulted in a notably increased 5-year OS (HR=0.53, 95% CI=0.27-1.01) and OS (HR=0.46, 95% CI=0.24-0.88) compared to CCRT alone (**Figures 2A, B**). However, no significant difference existed in the 2-year OS (HR=0.93, 95% CI=0.37-2.33) (**Figure 2C**).

### 3.3 Nomogram

**Figure 3:**
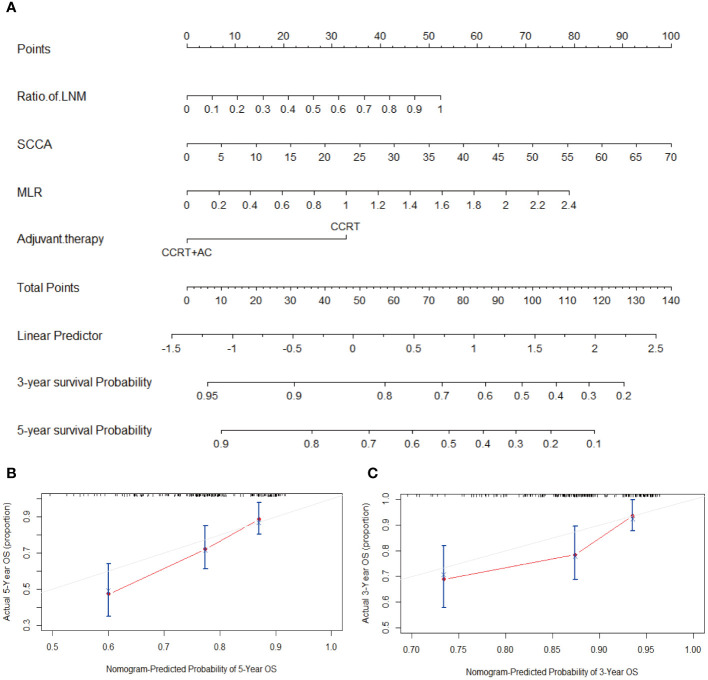
**(A)** Nomogram for predicting OS, which had a C-index of 0.75. **(B)** Calibration curve to predict 5-year OS. **(C)** Calibration curve to predict 3-year OS. Abbreviations: LNM, Lymph node metastasis; SCCA, squamous cell carcinoma antigen; MLR, monocyte-to-lymphocyte ratio; CCRT, concurrent chemoradiotherapy; CCRT+AC, concurrent chemoradiotherapy + adjuvant chemotherapy; OS, overall survival; C-index, concordance index.

To better predict the OS of patients with stage IIIC1p cervical cancer, nomograms were created using four factors: ratio of LNM, SCCA, MLR, and postoperative adjuvant therapy (CCRT or CCRT + AC) (**Figure 3A**). The C-index of the nomogram was 0.75, demonstrating strong concordance, as evidenced by the calibration curves for the 3 and 5-year periods depicted in **Figures 3B, C**.

## 4 Discussion

Cervical cancer primarily spreads through LNM, sequentially dispersing to the para-uterine, obturator, internal and external iliac, and common iliac lymph nodes. Approximately 10-20% of individuals diagnosed with cervical cancer in its early stages experience the spread of cancer cells to the lymph nodes (15). This study found that patients with postoperative pelvic LNM had a 5-year overall survival rate of 71.5%, which is consistent with the findings of previous studies. The 2018 FIGO staging system emphasizes the significance of lymph node status in prognosis and the recognition of diverse prognostic outcomes within this group.

The National Comprehensive Cancer Network (NCCN) guidelines recommend that patients with stage IB1- IIA2 cervical cancer with LNM receive platinum-based CCRT after surgery (16). Studies have shown that the postoperative recurrence and distant metastasis rates of patients with cervical cancer featuring LNM are significantly higher than those of patients without LNM (17). Fan et al. found that no postoperative adjuvant therapy was an independent risk factor for poor OS and DFS in patients with stage IIIC1p cervical cancer (9). Simultaneous dual-platinum-based chemotherapy and pelvic radiation therapy could be a potential approach to enhance results in these patients (18). Another potential strategy, augmenting CCRT with AC, did not show a significant difference in 5-year OS in the “Outback” trial (19). This is contrary to our findings where significant OS improvement was evident in the CCRT + AC group versus CCRT alone (p<0.05). These divergent outcomes could stem from the unique prognostic heterogeneity of stage patients with IIIC1p, which was not separately analyzed in the “Outback” trial. Therefore, our findings indicate that the CCRT + AC protocol could potentially enhance OS in individuals diagnosed with stage IIIC1p cervical cancer, highlighting the significance of this therapeutic strategy.

Several studies have confirmed that tumor-associated inflammatory cells are involved in tumor initiation and progression (20, 21). Fan et al. found that NLR>3.8 were independent risk factors for OS and DFS in patients with stage IIIC1p cervical cancer (9). Increased MLR indicated an increased in monocytes and a decrease in lymphocytes. This reflects a state of enhanced inflammation and reduced immunity, which favors tumor progression. Studies have shown that MLR is a valuable marker for the diagnosis and prognosis of various cancers including colorectal (22), breast (23), and gastric (24) cancers. According to our research, the MLR serves as a separate predictive element for individuals diagnosed with stage IIIC1p cervical cancer, and a higher preoperative MLR value corresponds to a poorer prognosis.

Yan et al. showed that the 5-year PFS and OS rates of patients with stage IIIC1p cervical cancer and > 2 LNM were significantly lower than those of patients with only 1-2 LNM (11). Our findings are similar to theirs. Our result shows that the p-value of > 2 LNM is significant in univariate but not in multivariate analysis. Theoretically, the number of LNM is directly influenced by the number of lymph nodes extracted. However, the ideal count for lymph node removals remains contentious (10, 25), and we believe that the quantity of LNM is an inadequate factor for forecasting the outlook of individuals diagnosed with stage IIIC1p cervical cancer. Fan et al. and Li et al. both analyzed the total number of lymph nodes resected, number of pelvic LNM, location of pelvic LNM, and ratio of LNM in patients with stage IIIC1p cervical cancer (9, 10). They identified the ratio of LNM >0.3 or the ratio of LNM ≥0.08 as an independent risk factor for OS and DFS in patients with stage IIIC1p cervical cancer, respectively, and conducted a more detailed stratified analysis of the ratio of LNM in the Li et al. study. The ratio of LNM has been acknowledged as a distinct prognostic indicator of different types of cancers (26–29). Some researchers have suggested that a higher ratio of LNM, is indicative of a higher intranodal tumor burden and may increase the likelihood of tumor cell spillage during lymphadenectomy, contributing to poorer outcomes (9). This hypothesis was consistent with our results. Therefore, we believe that the ratio of LNM holds significance in assessing the outlook of individuals diagnosed with stage IIIC1p cervical cancer.

Tumor markers are common indicators observed in patients with cancer to evaluate and monitor changes in their condition (30). Our research indicates that preoperative SCCA measurement serves as a stand-alone predictor in patients with stage IIIC1p cervical cancer. The nomogram showed that when the preoperative SCCA level was 70 ng/mL, the corresponding single score was 100. Furthermore, our study findings indicated that no significant correlation was evident between CEA levels and the prognosis of patients with stage IIIC1p cervical cancer. Studies have shown that CEA levels are an effective indicator of the prognosis of cervical adenocarcinoma (31, 32). We believe that this result is related to the fact that 81.7% of the pathologies in the collected samples were squamous cell carcinomas.

The incidence of common iliac LNM is lower in pelvic LNM of cervical cancer. Yan et al. found that the 5-year OS of patients with common iliac LNM was significantly lower than in those without common iliac LNM (11). However, common iliac LNM has no significant effect on OS in our study, and we believe that fewer patients with common iliac LNM is one of the reasons. There was research assessed the impact of the site of LNM in cervical cancer survival. Yan et al. found that > 2 LNM sites were predictive factors of poor survival in stage IIIC1p cervical cancer patients (11). Our result shows that LNM in two sites was strongly linked to OS. We think the difference in this result is related to our different classifications. In addition, we have an interesting finding that for patients who underwent PALN resection, although they did not have PALN metastasis, it still had an impact on the OS of stage IIIC1p cervical cancer patients. This is worth further research in the future.

Our study has several limitations. First, this was a retrospective study with all inherent limitations of this form of research. Second, the patients’ chemotherapy regimens were not uniform, and prospective studies are required to support our findings. Third, this study was conducted at a single medical facility with a limited number of participants, necessitating validation of the findings across numerous medical centers in future studies.

## 5 Conclusion

This study illustrates the heterogeneity in the prognosis of patients with stage IIIC1p cervical cancer. The ratio of LNM, SCCA, MLR, and CCRT + AC are independent prognostic predictors in patients with stage IIIC1p cervical cancer. In clinical practice, comprehensive evaluation of these factors is valuable for clinicians to treat and manage patients more proactively.

## Data availability statement

The original contributions presented in the study are included in the article/supplementary material. Further inquiries can be directed to the corresponding authors.

## Ethics statement

The studies involving humans were approved by First Affiliated Hospital of Guangxi Medical University Ethical Review Committee. The studies were conducted in accordance with the local legislation and institutional requirements. The ethics committee/institutional review board waived the requirement of written informed consent for participation from the participants or the participants’ legal guardians/next of kin because this is a retrospective study that only collects clinical information from patients.

## Author contributions

TG: Writing – original draft. ZY: Writing – original draft. LW: Formal analysis, Writing – original draft. XT: Formal analysis, Writing – original draft. SM: Formal analysis, Writing – original draft. LJ: Formal analysis, Writing – original draft. YZ: Writing – original draft. FW: Funding acquisition, Supervision, Validation, Writing – review & editing, Writing – original draft.
